# HN1L is essential for cell growth and survival during nucleopolyhedrovirus infection in silkworm, *Bombyx mori*

**DOI:** 10.1371/journal.pone.0216719

**Published:** 2019-05-22

**Authors:** Jihai Lei, Dongbing Hu, Shengjie Xue, Fuxiang Mao, Enoch Obeng, Yanping Quan, Wei Yu

**Affiliations:** 1 Institute of Biochemistry, College of Life Sciences and Medicine, Zhejiang Sci-Tech University, Hangzhou, Zhejiang, People’s Republic of China; 2 Zhejiang Provincial Key Laboratory of Silkworm Bioreactor and Biomedicine, Hangzhou, Zhejiang, People’s Republic of China; Duke University School of Medicine, UNITED STATES

## Abstract

Hematological and neurological expressed 1-like (HN1L) protein is an evolutionarily conserved protein that plays an important role in embryonic development. It has been reported that HN1L is involved in the process of cell growth and cancer formation and that cell cycle arrest occurs during suppression of HN1L expression. Previous studies have demonstrated that the expression levels of the *Bombyx mori* HN1L protein were significantly downregulated in *Bombyx mori* Nucleopolyhedrovirus (BmNPV) infected silkworm cells. Transient transfections were performed with plasmids for pIEX-1-HN1L expression in *Bombyx mori* ovarian cells (BmN) in order to explore the effect of the HN1L protein on the growth of silkworm cells and its regulatory role in the process of viral infection. Cellular localization analysis revealed that HN1L was localized in the cytoplasm and that its upregulation could significantly enhance cellular activity. Furthermore, HN1L could promote G1/S phase conversion, thereby contributing to cell proliferation. Upon infection of BmN cells with BmNPV, the induction of apoptosis increased, although HN1L overexpression could inhibit DNA fragmentation, suggesting that the HN1L protein could inhibit cell apoptosis induced by viral invasion. In addition, Western blotting indicated that the HN1L protein inhibited the activation of caspase-9 zymogen and the expression of Bax protein, although it promoted Bcl-2 expression. Flow cytometry analysis further confirmed that overexpression of HN1L significantly inhibited apoptosis induced by BmNPV infection. Consequently, we demonstrated that BmN HN1L is a protein with multiple functions, which enhanced cell activity, regulated the cell cycle and induced an anti-apoptotic response by BmNPV infection.

## Introduction

Silkworm is an important lepidopteran model organism with economic significance for the production of silk and the expression of proteins used in the pharmaceutical industry [1–3]. *Bombyx mori* Nucleopolyhedrovirus (BmNPV) is a pathogenic virus that specifically infects silkworms and causes serious larval death and large economic loss to the sericulture [4]. During viral infection, a wide interaction occurs between the host and the virus. In addition, the host changes its own metabolism to respond to the viral invasion. It has been reported that the enzyme activity of alkaline phosphatase in *Podoptera litura* decreased following *Nucleopolyhedrovirus* (NPV) infection [5]. In addition, alkaline phosphatase enzyme activity in the silkworm embryo cells declined following BmNPV infection, whereas the levels of the endogenous compounds cholesterol, urea and glucose were also significantly reduced [6]. In addition, it was shown that the total levels of the hemolymph protein of the viral-infected Lepidoptera larvae were reduced compared with those of the uninfected larvae, although the activities of the two types of aminotransferases were significantly increased [7]. The data indicated that viral infection exhibited a significant effect on cell metabolism. We have previously shown that BmNPV infection causes significant changes in the proteome and acetylome of BmN cells [8]. A total of 33 proteins were upregulated and 47 proteins were downregulated in the total 4,194 host proteins quantified. Among these proteins HN1L exhibited significantly higher differences in expression following BmNPV infection.

Hematological and neurological expressed 1 (*HN1*) was initially isolated from a cDNA library of fetal murine tissues and was named according to the highest levels of expression in hemopoietic cells and fetal brain [9]. It was postulated that HN1 is involved in embryo development, notably in the proliferation and differentiation of hemopoietic and neurological cells. The homologous form of *HN1* with high N-terminal homology is called *Hn1-like* (*HN1L*) and encodes the HN1L protein with a molecular mass of 20.9 kD. Similarly, HN1L exhibits potential roles on embryonic development. No conserved domains have been identified that are similar to HN1 or the HN1L proteins. Molecular evolution analysis revealed that *HN1* and *HN1L* belong to larger conserved multigene protein families [10]. HN1 and HN1L are highly conserved among species and are expressed in a variety of tissues important for cell development. It has been reported that the HN1 protein is highly expressed in the immature newt retinas, and that it is an important factor for inducing reconstruction of newt neural retinas [11]. However, *HN1* silencing in melanoma cells causes accumulation of cells at the G1/S phase and Cyclin D protein overexpression [12]. In addition, HN1L regulates multiple signaling pathways in order to maintain tumor survival, including STAT3 signaling that is closely associated to cell growth, differentiation and apoptosis [13]. In addition, *HN1L* silencing further reduces the CSC population in TNBC cell lines and depresses the development of tumors [13]. This evidence indicated that HN1 and HN1L proteins act as regulators of signaling pathways and play important roles in cell growth and development via modulating cell cycle and apoptosis. However, in silkworm the function of HN1 and HN1L proteins has not been well characterized.

In the present study, we described the potential impact of HN1L on BmN cell growth and explored its mechanism of action. In addition, we provide a new potential mechanism that involves cell survival regulation by HN1L via BmNPV infection. To this end, a transient plasmid pIEX-1-*HN1L* was constructed and transfected into BmN cells. Cell viability assay demonstrated that HN1L promoted cell proliferation. The examination of the cell cycle proteins demonstrated that HN1L upregulation decreased the levels of Cyclin D expression and the ratio of cells at the G1 phase. However, the ratio of the cells in the S phase was increased. The data revealed that HN1L protein promoted cell proliferation by facilitating the transition of the cells from the G1 to the S phase by depletion of Cyclin D. In contrast to these observations, the potential role of maintaining high cell growth activity was verified by BmN cells that were infected with BmNPV. Upon infection, the virus induced BmN cell rupture and cell death. HN1L protein exhibited an anti-apoptotic role in order to maintain cell survival by regulating the expression of the apoptosis-related proteins Bax and Bcl-2, as well as the activation of caspase-9.

## Materials and methods

### Cells and virus

BmN cells that were derived from silkworm ovary were cultured at 27°C in Sf-900 medium (Thermo Fisher Scientific, America) supplemented with 10% fetal bovine serum (Corning, America). The BmNPV genome was extracted from the *E*. *coli* strain DH10Bac and transfected into BmN cells to propagate the virus. Viral titers were determined by end-point dilution assay (Reed and Muench, 1938) [14].

### Plasmid construction

Total RNA from BmN cells was extracted by Trizol reagent (Pufei Biotech, Shanghai, China) according to the manufacturer’s recommendations. The single-strand cDNA was carried out using the 1st strand cDNA synthesis kit (Thermo Fisher Scientific, MA, USA). Based on the *HN1L* sequence, specific primers were designed in order to amplify the gene sequence with relevant restriction sites for the enzymes *Bam*H and *Xho*. The *HN1L* fragments were amplified by PCR, digested by *Bam*H and *Xho* and then ligated to the *Bam*H/*Xho*-digested pIEX-1 vector, which contained His-tag. This resulted in the production of the overexpression plasmid pIEX-1-*HN1L*. Similarly, the recombinant plasmid pIEX-1-*HN1L*-eGFP was constructed. All clones were confirmed by restriction enzyme digestion and nucleotide sequence analysis.

### HN1L expression

BmN cells were plated in 35 mm-diameter dishes at a concentration of 1×10^6^ cells/well and were transfected with 1.0 μg endotoxin-free pIEX-1-*HN1L*-eGFP plasmids using transfection reagents (Pufei Biotech, Shanghai, China). Following 4 h of incubation, the transfection reagent was replaced with fresh culture medium. BmN cells were harvested at the indicated times post-transfection. The expression levels of *HN1L* were evaluated by Western blot analysis using rabbit polyclonal anti-HN1L antibody that was made by HuaAn Biotechnology company (Hangzhou, China).

### Subcellular localization

BmN cells (3×10^4^ cells per 35 mm-diameter dish) were transfected with 2.0 μg pIEX-1-*HN1L* and pIEX-1-*HN1L-eGFP*, respectively. At 48 h post-transfection, the cells were harvested, or infected with BmNPV for 24 hours, respectively. The infected or uninfected cells transfected with pIEX-1-*HN1L* were washed thrice with PBS and fixed with 4% paraformaldehyde in PBS. The cells were permeabilized using 0.5% TritonX-100 for 20 min and incubated with a sealing fluid for 1 h at 37 °C. The cells were incubated with affinity purified rabbit polyclonal anti-HN1L and anti-His antibodies at 1:100 for 1 h at room temperature. Following the incubation, the cells were rinsed in PBS three times for 10 min. The anti-rabbit fluorescent secondary antibody (green) (Multi Sciences, Hangzhou, China) and the Alexa Fluor 555-Labeled Donkey Anti-Rabbit IgG (red) (Beyotime, Shanghai, China) were used at 1:500 for 1 h at room temperature. For nuclear staining, the cells were counterstained with DAPI. Finally, the cells were visualized under a fluorescence microscope. The cells with the GFP fusion protein were visualized directly under a confocal microscope following nuclear staining with DAPI.

### Cell proliferation assay

BmN cells were seeded in 96-well plates at a density of 5×10^3^ cells per well and transfected with pIEX-1-*HN1L* plasmids at a concentration of 0.4, 0.2, 0.1, 0.05, 0.01 or 0.001 μg/well, respectively. The pIEX-1 plasmids were also incorporated in BmN cells as a control. Each dilution was repeated five times. Following 48 h of incubation, fresh culture medium was added to the cells, which contained 10% CCK-8 reagent (Dojindo Molecular Technologies, Japan). The cells were incubated for 6 h at 27 °C and the absorbance was read at 450 nm with a Microplate Reader.

### Western blotting analysis

BmN cells were transferred in 35-mm dishes at a concentration of 1×10^6^ cells per dish and were transfected with 1.0 μg of pIEX-1-*HN1L* and pIEX-1, respectively. At 48 h post-transfection, the cells were harvested together with normal BmN cells. Furthermore, the normal cells and the test group cells which had been transfected with 1.0 μg of pIEX-1-*HN1L* for 48 h, were infected with BmNPV at a multiplicity of infection of 10. At 6, 12, 24 and 36 h post-infection, the cells were harvested respectively. All the aforementioned cell samples were rinsed twice with PBS and lysed with 100 μl lysis buffer (50 mM Tris pH 7.5, 150 mM NaCl, 1 mM EDTA, 0.5% NP-40) that contained protease inhibitors (Bimake, TX, USA). The proteins were then collected by centrifugation at 14,000×g for 15 min at 4°C and resolved by 12% sodium dodecyl sulfate-polyacrylamide gel electrophoresis, followed by electroblotting to PVDF membranes. The mouse monoclonal anti-cyclin D1 antibody (Santa Cruz Biotechnology, TX, USA) and the mouse polyclonal anti-caspase-9 antibody (Cell Signaling Technology, MA, USA) were used at 1:1,000 dilutions and the secondary antibody, namely HRP-conjugated sheep anti-mouse (Saiguo Biotech, Guangzhou, China) antibody, was used at 1:5,000 dilution. In addition, the mitochondrial proteins were isolated using the cytoplasmic and mitochondrial protein extraction kit (Sangon Biotech, Shanghai, China), followed by immunoblotting analysis with mouse monoclonal anti-Bax and anti-Bcl-2 antibodies (1:1,000, ImmunoWay Biotechnology, America). Immunoblotting analysis was performed using the chemiluminescent reagents (Cell Signaling Technology, America).

### Flow cytometry analysis

The cells were seeded on 6-well plates at a density of 1×10^6^ cells per well and transfected with 1.0 μg of pIEX-1-*HN1L* plasmids. At 48 h post-transfection, the cells were infected with BmNPV at a M.O.I. of 10. Similarly, the normal cells were also infected with BmNPV. At 24 h post-infection, the cells were collected and counted by flow cytometry using a cell cycle staining kit (Multi Sciences, Hangzhou, China) and an Annexin V-FITC/PI apoptosis assay kit (Multi Sciences, Hangzhou, China) which were used in accordance with the manufacturer’s instructions. The non-infected cells that were transfected, or not transfected with plasmids were designed as controls.

### DNA fragmentation assay

The BmN cells were transfected with 1 μg of pIEX-1-*HN1L* plasmids and infected with BmNPV as described previously. At 24 h post-infection, the cells were collected and counted. The DNA fragmentation index of the cells (1×10^4^) from each sample was detected using the Cell Death detection ELISA kit (Roche, Basel, Switzerland) according to the instructions provided by the manufacturer.

## Results

### Expression and localization of HN1L protein in BmN cells

**Fig 1:**
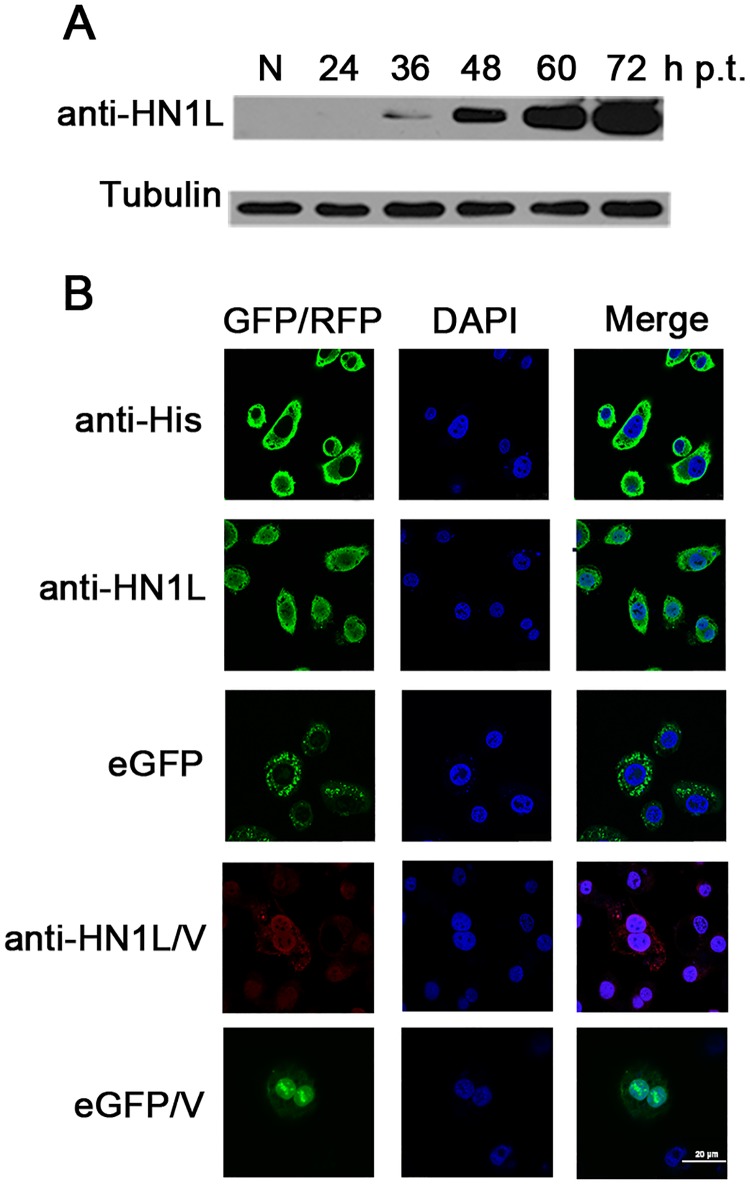
Expression and localization of HN1L protein in BmN cells. (A) BmN cells were transfected with the plasmids pIEX-1-*HN1L*-eGFP. At 24, 36, 48, 60 and 72 h post-transfection, the cells were harvested and detected by Western blotting using the anti-HN1L antibody. Normal cells were designated as control (N), whereas tubulin was employed as internal reference. (B) BmN cells were transfected with the plasmids pIEX-1-*HN1L* and pIEX-1-*HN1L*-eGFP, respectively. At 48 h post-transfection or 24 h post-infection, the cells were fixed or examined directly for eGFP fusion expression (GFP). The fixed cells were stained with a rabbit polyclonal anti-HN1L antibody (1:100) and a rabbit polyclonal anti-His antibody (1:100), and then detected with an anti-rabbit fluorescent secondary antibody (1:500) (GFP/RFP). The cells were also stained with DAPI for nuclear detection. The combination of the GFP/RFP and the DAPI images (Merge) revealed the subcellular localization of the HN1L protein.

In order to investigate the expression levels of the HN1L protein in BmN cells cultured *in vitro*, Western blotting was carried out to detect the HN1L protein using rabbit polyclonal anti-HN1L antibody. The HN1L protein was detected at 36 h following transfection, and its levels were increased significantly at 48 h post-transfection, followed by a stationary period (Fig 1A). Therefore, 48 h post-transfection was selected as the optimal time point for the following experiments.

The subcellular distribution of the HN1L protein was investigated by the immunofluorescence assay. BmN cells were transfected with pIEX-1-*HN1L* and pIEX-1-*HN1L-eGFP*, which resulted in HN1L protein fusion with His-tag or eGFP proteins, respectively. At 48 h post-transfection or 24 h post-infection, the cells were fixed and immunostained, and then scanned using confocal microscopy. The immunofluorescence assay results indicated that the HN1L protein was originally located in the plasma membrane and the cytoplasm of BmN cells (Fig 1B), which was consistent with the distribution pattern of HN1L-eGFP observed by its autofluorescence. However, HN1L showed nuclear and cytoplasmic distribution after BmNPV infection in BmN cells (eGFP/V, anti-HN1L/V), and the nuclear fluorescent signals are stronger than cytoplasmic signals.

### HN1L upregulation facilitates cell proliferation *in vitro*

**Fig 2:**
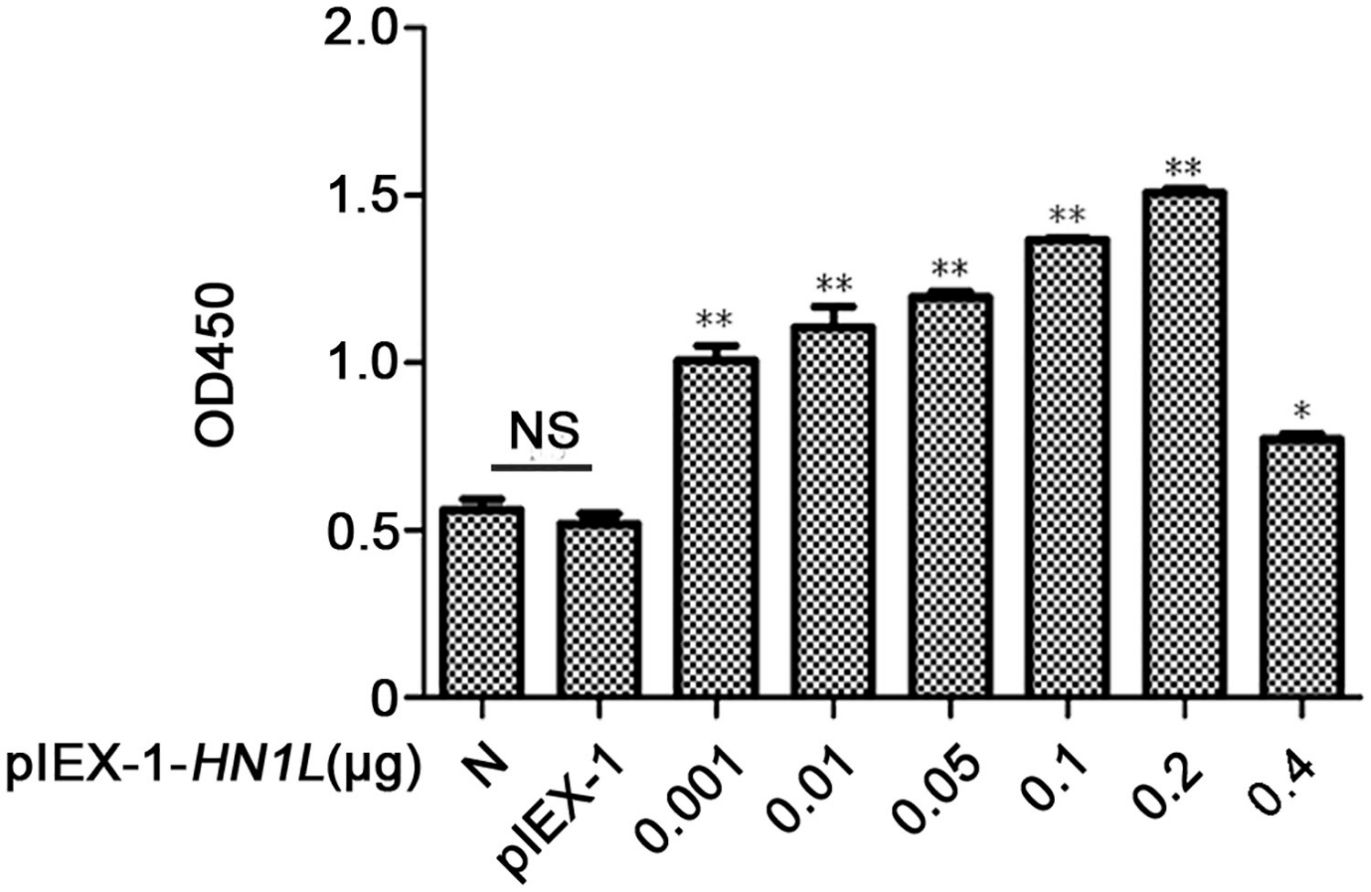
HN1L upregulation facilitates cell proliferation *in vitro*. BmN cells were transfected with pIEX-1-*HN1L* plasmids at a concentration of 0.4, 0.2, 0.1, 0.05, 0.01 or 0.001 μg per well. Both normal cells (N) and the cells transfected with empty vectors (pIEX-1) were used as a control cells. Following treatment with 10% of CCK-8 reagent, the OD_450_ value of BmN cells was measured by a microplate reader. No significant differences were noted between normal cells and the cells transfected with the pIEX-1 plasmid. However, the difference between normal cells and the cells transfected with the pIEX-1-*HN1L* plasmid was statistically significant (NS, not significant, * *P* < 0.05, ** *P*<0.01). The error bars indicate standard deviations of the mean (n = 3).

BmN cells were transfected with various concentrations of pIEX-1-*HN1L* plasmids in order to study the effects of HN1L on cell activity. The OD_450_ value of pIEX-1-transfected cells increased significantly as the concentration of plasmid increased compared with the normal cells (Fig 2). The results suggested that the upregulation of HN1L protein levels enhanced the cellular activity and promoted cell proliferation.

### Overexpression of HN1L promotes the transition from G1 to S phase

**Fig 3:**
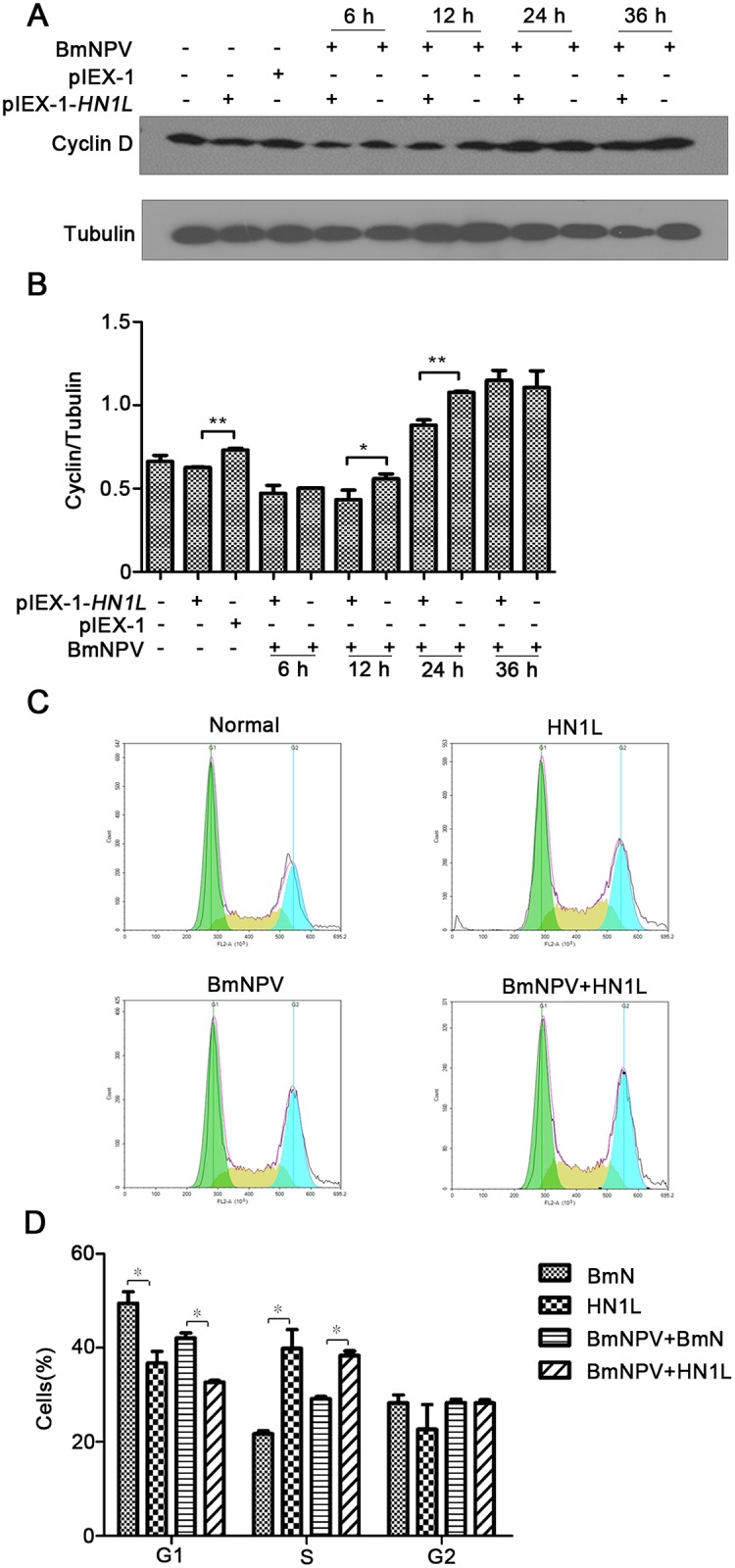
Overexpression of HN1L promotes the G1 to S phase transition. (A) The normal cells and the cells which had been transfected with 1.0 μg of pIEX-1-*HN1L* for 48 h, were infected with BmNPV at a multiplicity of infection of 10. The cells were harvested and protein expression was detected by Western blotting at specific time points. The mouse monoclonal anti-cyclin D1 antibody was used at a 1:1,000 dilution and tubulin was selected as an internal reference. (B) The difference of cyclin D expression was analyzed by semi quantitative methods (*P < 0.05, ** *P*<0.01). (C) The effect of different treatments on the distribution of the cells during different phases of the cell cycle was analyzed by flow cytometry. The normal cells (Normal) and the cells infected by BmNPV (BmNPV) were denoted as controls. The HN1L-overexpressing cells (HN1L) and that infected with BmNPV (HN1L+BmNPV) exhibited increased number in the S phase of the cell cycle. (D) Significant differences in the population of the cells in the various cell cycle phases following overexpression of HN1L. The error bars indicate standard deviations of the mean values. * *P* < 0.05.

To further investigate the role of HN1L on cell growth during BmNPV infection, cell cycle analysis was carried out. It has been reported that downregulation of HN1L causes cyclin D accumulation and G1/S cell cycle arrest [12]. In the present study, the differences in cyclin D levels in BmN cells that overexpressed HN1L were detected (Fig 3A). Semi-quantification analysis of cyclin D expression levels in HN1L overexpressing cells demonstrated that they were significantly lower compared with those of the control cells without overexpression of HN1L at the same time point (Fig 3B). Similar results were obtained following BmNPV infection, whereas from 24 h post-infection, the total cyclin D levels increased. In addition, overexpression of HN1L resulted in significantly lower number of cells at the G1 phase and a significant increase in the proportion of cells at the S phase compared with the corresponding ratios in the control cells (Fig 3C and 3D). The results revealed that HN1L promoted the transition from G1 to S phase of the cell cycle.

### HN1L is important for cell survival following BmNPV challenge

**Fig 4:**
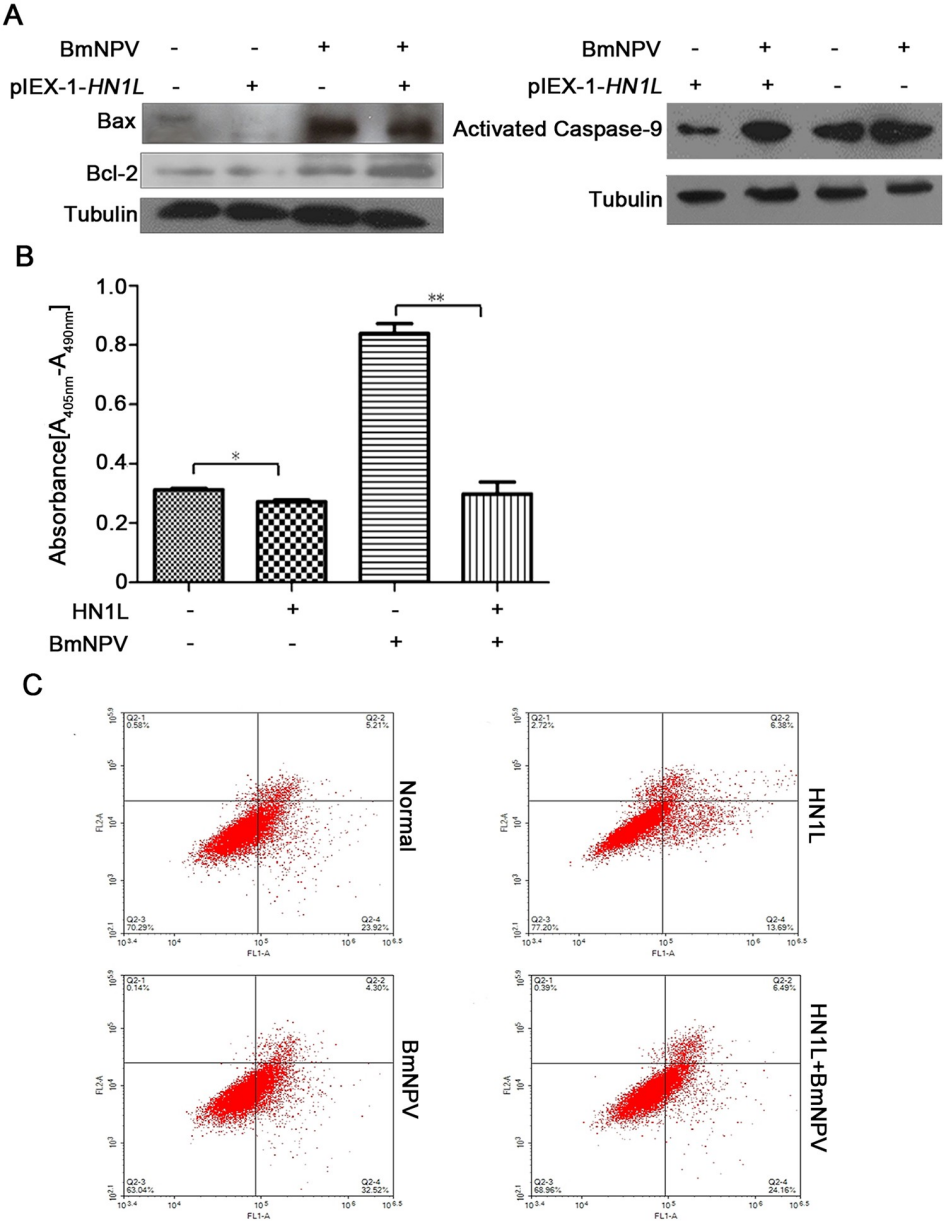
HN1L is important for cell survival following BmNPV challenge. (A) BmN cells were harvested at 24 h post-infection and the mitochondrial proteins were isolated. Alterations in the expression levels of Bax, Bcl-2, and caspase-9 were detected by immunoblotting using the mouse monoclonal anti-Bax and anti-Bcl-2 antibodies and the mouse polyclonal anti-caspase-9 antibody. (B) The effects of overexpressing HN1L on DNA fragmentation of viral-infected BmN cells or non-infected BmN cells. The error bars indicate standard deviations of the mean values. * *P* < 0.05, ** *P*<0.01. (C) Effects of overexpressing HN1L on the induction of apoptosis, as demonstrated by flow cytometry.

Having shown that HN1L benefits cell survival and growth, the effects of HN1L on the induction of cell apoptosis by BmNPV infection were explored. BmN cells that overexpressed HN1L were infected with BmNPV and harvested at 24 h post-infection. Western blotting demonstrated that Bax, caspase-9, and Bcl-2 protein levels were increased compared with those noted in normal cells, which were not infected by the virus (Fig 4A). However, caspase-9 and Bax protein levels declined and Bcl-2 protein levels were increased in HN1L overexpressing cells compared with the cells that did not overexpress HN1L, but were infected by BmNPV. The results demonstrated that BmNPV infection induced apoptosis, whereas HN1L could inhibit this process. ELISA assays confirmed that DNA fragmentation levels were significantly reduced in cells that overexpressed HN1L during viral infection (Fig 4B). In addition, flow cytometry results indicated that the apoptotic rate in cells that overexpressed HN1L was lower than that of the control cells (Fig 4C). Taken collectively, the data demonstrated that HN1L inhibits apoptosis induced by BmNPV infection in order to support cell survival.

## Discussion

*HN1* and *HN1L* are members of a new large gene family, and play important roles for normal cell growth and development. Their upregulated expression levels are crucial for the maintenance of tumor survival. The depletion of HN1 or HN1L results in cell cycle arrest and consequently suppression of cellular proliferation in different types of cancer [12][13][15]. *HN1* has been described as an oncogene and is a therapeutic target, as the inhibition of its expression can restrict breast cancer cell proliferation, invasion, migration and metastasis [16]. This evidence has demonstrated that HN1 and HN1L play an important role in oncogenesis and in cell development and proliferation.

The completion of the silkworm genome project [17] enabled the detailed analysis of the mechanism of interaction between the silkworm and the baculovirus. We previously demonstrated by LC-MS/MS analysis that the HN1L protein was downregulated by 30% following viral infection [8]. HN1L in *Bombyx mori* shares 59% homology between the human and mouse genomes, whereas a common conserved domain in the N-terminal region of the protein exists. Thus, HN1L in silkworm may play a similar role in the regulation of cell proliferation and apoptosis. In addition, the data reported in the present study revealed that downregulation of HN1L might function as a cellular defense against early viral invasion required for cell survival, although the underlying mechanism remains unclear.

The subcellular localization results revealed that HN1L was mainly localized in the plasma membrane and the cytoplasm of BmN cells, which implies that HN1L is not a terminal factor directly involved in regulating gene expression in the nucleus. This is in accordance with other reports, which demonstrated that HN1L plays a crucial role in promoting multiple cellular survival pathways in breast cancer stem cells [13]. However, the viral infection resulted in typical nucleocytoplasmic localization of HN1L, particularly showing distinct cytoplasm-to-nucleus redistribution, which implies that the HN1L is involved in the interaction between silkworm and BmNPV. It is likely that HN1L underwent some certain process during virus invasion, resulting in the change of the subcellular localization.

HN1 is involved in the regulation of the AKT/GSK3β/β-catenin pathway. Alterations in the levels of of β-catenin expression can affect the expression levels of the downstream gene *cyclin D*, which is associated with cell cycle and cell proliferation [13][18][19]. Furthermore, the data indicated that the overexpression of HN1L significantly enhanced cell viability and promoted cell proliferation. Furthermore, we demonstrated that cyclin D protein levels were significantly lowered in cells that overexpressed HN1L, although in the late stages of viral infection, the levels of cyclin D protein were increased due to the induction of apoptosis caused by the mature virus. In addition, flow cytometry results further confirmed that HN1L promoted BmN cells from the G1 to the S phase, even in the early stages of viral invasion. The results demonstrated that HN1L promoted cell proliferation via enhancing the transition of G1 to S phase by the depletion of the cyclin D protein. It is possible that HN1L functions in a similar manner with HN1, and that it involves the AKT/GSK3β/β-catenin signaling pathway. However, further research studies are required for the exploration of the underlying mechanism.

In addition to the regulation of the cell cycle, HN1L may also participate in other pathways to maintain high cell survival and high proliferative activity. The induction of PC12 (nerve cells) apoptosis that was accomplished by ceramide treatment caused a downregulation of HN1 expression that was 3-fold lower compared with the expression noted in the control samples [20]. Similarly, when BmN cells were infected by BmNPV, the expression of HN1L was significantly downregulated. The data demonstrated that HN1 and HN1L were involved in the apoptotic-signaling pathway and that they might interfere with the occurrence of apoptosis. The infection of normal BmN cells with BmNPV caused high-levels of DNA fragmentation and this was significantly inhibited in cells that overexpressed HN1L. The results preliminarily confirmed that inhibition of HN1L inhibited the induction of apoptosis. Furthermore, the flow cytometry results confirmed that the overexpression of the HN1L protein reduced the number of cells that could undergo apoptosis.

However, the exact mechanism by which HN1L inhibits apoptosis is unknown. In the present study, we further explored the mechanism of HN1L regulation on the induction of apoptosis. Bcl-2 and Bax proteins are two critical mediators that regulate mitochondrial membrane permeability in the caspase-dependent endogenous apoptotic pathway [21][22]. Moreover, caspase-9 is an indispensable apoptosis factor. We investigated the alterations in the expression levels of this critical apoptotic protein. As expected, the overexpression of HN1L caused a variation in the expression levels of Bax, Bcl-2 and caspase-9 proteins in BmN cells, which was in accordance with the inhibition of cell apoptosis. The expression of the pro-apoptotic protein Bax was downregulated and the levels of the Bcl-2 protein were raised. The activation of the caspase-9 protein was inhibited in cells that overexpressed HN1L. This demonstrated that HN1L was involved in an endogenous apoptotic pathway via regulating the expression levels of the apoptosis-related proteins. However, additional studies are required to elucidate the exact mechanism of action of HN1L in the induction of apoptosis.

In the present study, we demonstrated that HN1L was involved in cell cycle regulation and apoptosis and that it promoted cellular growth and proliferation. HN1L facilitated the G1/S phase transition that was associated with cyclin D depletion. In addition, HN1L inhibited the induction of apoptosis by viral invasion through regulating the levels of the apoptosis-related proteins, notably Bax, Bcl-2 and activated caspase-9. Taken collectively, the data indicated that HN1L played an important role in the regulation of both cell cycle and apoptosis in order to maintain high cell survival and proliferative activity. These findings further elucidate the function of HN1L and the mechanism of its action, and provide new insights into the mechanism of interaction between the silkworm and BmNPV.

## Supporting information

S1 Data Data file. (DOCX)
