# Supplementary material for: HN1L is essential for cell growth and survival during nucleopolyhedrovirus infection in silkworm, Bombyx mori
Source: PLoS One. 2019 May 22;14(5):e0216719. doi: 10.1371/journal.pone.0216719 (PMC6532551; doi:10.1371/journal.pone.0216719)
Supplement: S1 Data — (DOCX) [file pone.0216719.s001.docx]

Figure 2. HN1L upregulation facilitates cell proliferation in vitro

| plasmid | pIEX-1-*HN1L*(μg) | | | | | | pIEX-1 | Normal |
| --- | --- | --- | --- | --- | --- | --- | --- | --- |
|  | 0.4 | 0.2 | 0.1 | 0.05 | 0.01 | 0.001 |  |  |
| OD450 | 0.774 | 1.515 | 1.376 | 1.218 | 1.051 | 1.012 | 0.508 | 0.519 |
| OD450 | 0.743 | 1.488 | 1.357 | 1.162 | 1.04 | 0.931 | 0.576 | 0.543 |
| OD450 | 0.799 | 1.522 | 1.37 | 1.207 | 1.228 | 1.079 | 0.474 | 0.621 |
| average | 0.772 | 1.508333 | 1.367667 | 1.195667 | 1.106333 | 1.007333 | 0.519333 | 0.561 |

Fig 4B. The effects of overexpressing HN1L on DNA fragmentation of viral-infected BmN cells or non-infected BmN cells

|  | OD405 | OD490 | OD405-OD490 |
| --- | --- | --- | --- |
| HN1L | 0.357 | 0.073 | 0.284 |
|  | 0.33 | 0.064 | 0.266 |
|  | 0.343 | 0.065 | 0.278 |
| HN1L/BmNPV | 0.409 | 0.07 | 0.339 |
|  | 0.325 | 0.066 | 0.259 |
|  | 0.342 | 0.056 | 0.286 |
| Normal | 0.385 | 0.068 | 0.317 |
|  | 0.377 | 0.068 | 0.309 |
|  | 0.393 | 0.077 | 0.316 |
| Normal/BmNPV | 0.988 | 0.116 | 0.872 |
|  | 0.916 | 0.111 | 0.805 |
|  | 0.911 | 0.086 | 0.825 |
